# Evaluation of micro-gap at implant abutment interface using different connection design

**DOI:** 10.6026/973206300222283

**Published:** 2026-04-30

**Authors:** Vidhyadhar D, Tejosmita Chowdary Pavuluri, Vivek Anne, Sruthi Katamneni, B. Vimal Bharathi, Radha Chiluka, Sriharsha Pudi

**Affiliations:** 1 Department of Prosthodontics Crown and Bridge, Balaji Family Dental Care, Kamareddy, Telangana, India; 2 Department of Biomedical Sciences, Rutgers Biomedical and Health Sciences, Newark, New Jersey, 07107, USA; 3 Department of General Dentistry, Always Smile Dental Care, North Brunswick Township, New Jersey, USA; 4 Department of General Dentistry, Indiana University School of Dentistry Indianapolis, Indiana, USA; 5 Department of Prosthodontics, Panineeya Institute of Dental Sciences, Hyderabad, Telangana, India; 6 Department of Prosthodontics, MNR Dental College and Hospital, Sangareddy, Telangana, India

**Keywords:** Abutment interface, cyclic loading, implant connection, micro-gap, scanning electron microscope (SEM)

## Abstract

The persistent presence of a micro-gap at the implant-abutment interface continues to pose biological and mechanical challenges, yet the influence of different connection designs remains insufficiently understood. Therefore, it is of interest to evaluate 100 implant abutment assemblies external hex, internal hex and internal conical using SEM before and after cyclic loading. External hex connections showed the largest micro-gaps, internal hex showed moderate values and internal conical exhibited the smallest and most stable interface. Statistical analysis confirmed significant differences among the designs, with conical connections demonstrating superior resistance to micro-gap enlargement under functional loading. Thus, we show new comparative evidence showing that internal conical connections provide the most stable interface, thereby advancing knowledge on optimal implant-abutment design for improved clinical outcomes.

## Background:

Dental implants have become a predictable and widely accepted treatment modality for the replacement of missing teeth. Their long-term clinical success depends not only on osseointegration but also on the stability and precision of the implant-abutment interface [1]. The implant-abutment junction represents a critical biomechanical and biological zone, where the design and accuracy of the connection play a significant role in overall implant performance [2]. One of the most important concerns in this area is the formation of a micro-gap, which refers to a microscopic space that can exist between the implant fixture and the abutment even when both components appear clinically well-seated [3]. The presence of a micro-gap has multiple implications. It can serve as a reservoir for bacterial colonization, allowing microorganisms and their by-products to penetrate the internal compartment of the implant. This bacterial infiltration can trigger an inflammatory cascade leading to peri-implant mucositis and, over time, peri-implantitis and crestal bone loss [4]. Numerous studies have shown that the micro- gap acts as a "pump effect", whereby functional loading and micro-movement at the interface force bacteria in and out of the gap, amplifying microbial contamination. Thus, the biological seal around the implant is compromised when gaps exceed certain thresholds [5]. In addition to biological concerns, the micro-gap also has mechanical consequences. Micromotion at the interface may result in screw loosening, abutment instability, or even component fracture over prolonged function. These mechanical complications negatively affect implant longevity and often require additional interventions. Therefore, reducing micro-gap formation is a key objective in modern implant engineering [6]. Over the years, different implant-abutment connection designs have been introduced to improve stability and minimize microleakage. Broadly, these include external hex, internal hex, internal conical (Morse taper), platform- switched and hybrid connections. External hex connections, although historically popular, are more susceptible to micro-movement and screw loosening [7]. Internal hex designs provide improved anti-rotational stability but may still exhibit measurable micro-gaps under functional loading. Conical or Morse taper connections have shown promise in creating a "cold-weld" effect, thereby significantly reducing micro-gap size and bacterial penetration. Platform switching, where the abutment is narrower than the implant platform, further shifts the inflammatory zone away from crestal bone, improving marginal bone preservation [8]. Understanding micro-gap behavior across different connection designs is essential for improving implant longevity, reducing complication rates and guiding clinicians in evidence-based implant selection. As implant dentistry continues to evolve, the need for precise, well- engineered interfaces becomes even more important for long-term clinical success [9]. Therefore, it is of interest to determine the extent of micro-gap formation among different implant-abutment connection designs, compare their biological and mechanical implications and identify which configuration offers the most stable and clinically favorable interface for long-term implant success.

## Methodology:

This *in vitro* study was conducted to evaluate the micro-gap at the implant-abutment interface among different implant connection designs. A total of 100 implant fixtures were selected and divided into groups based on connection type, including external hex, internal hex and internal conical (Morse taper) connections. All implants used in the study were of the same manufacturer to ensure uniformity in material composition and machining tolerances. Corresponding abutments compatible with each connection type were obtained and all specimens were handled according to the manufacturer's guidelines. Each implant fixture was mounted vertically in acrylic resin blocks using a custom-made jig to ensure standardized positioning during testing. Abutments were then connected to the implants using a calibrated torque wrench, applying the recommended torque value. After initial tightening, all samples underwent a 10-minute settling period, following which the torque was reapplied to account for torque loss due to embedment relaxation. This ensured consistent and clinically relevant preload across all specimens. Once assembled, the implant-abutment complexes were subjected to micro-gap evaluation. Samples were analyzed using a high-resolution scanning electron microscope (SEM) at magnifications ranging from 200x to 1000x. The implant-abutment junction was examined at four standardized points-buccal, lingual, mesial and distal-and the vertical micro-gap was measured using digital imaging software calibrated with a precision scale. For each sample, the mean of the four measurements was recorded as the representative micro-gap value. To assess the effect of functional loading on micro-gap formation, the samples were placed in a cyclic loading machine and subjected to mechanical loading, simulating masticatory forces. Following cyclic loading, SEM evaluation was repeated using the same protocol to determine any changes in the micro-gap dimension. All collected data were tabulated and subjected to statistical analysis. Normality of data distribution was assessed using the Shapiro-Wilk test. Comparisons between groups were performed using one-way analysis of variance (ANOVA), followed by post hoc tests for intergroup comparison where applicable. A significance level of p < 0.05 was considered statistically meaningful. This standardized methodological approach ensured reproducibility of measurements, minimized operator- induced variability and allowed reliable comparison of micro-gap formation among different implant-abutment connection designs.

## Results:

A total of 100 implant-abutment assemblies were evaluated and divided into three groups based on connection design: External Hex (n = 34), Internal Hex (n = 33) and Internal Conical/Morse Taper (n = 33). All samples were successfully examined under SEM before and after cyclic loading. The mean micro-gap values varied significantly among the connection systems. Initial SEM measurements demonstrated the highest micro-gap values in the external hex group, followed by internal hex, whereas the internal conical connection exhibited the smallest micro- gap dimension (Table 1 - see PDF). After cyclic loading, all groups showed an increase in micro-gap values; however, the increase was least evident in the internal conical group (Table 2 - see PDF). Intergroup comparison using one-way ANOVA showed statistically significant differences between the three connection types both before and after mechanical loading (p < 0.001). Post-hoc analysis revealed that the internal conical connection had significantly lower micro-gaps when compared with both external and internal hex groups (Table 3 - see PDF). Paired comparison within each group revealed a significant increase in micro-gap after loading (p < 0.05), with the external hex group demonstrating the highest change, followed by internal hex and the least change observed in the internal conical connection (Table 4 - see PDF). These findings indicate superior mechanical stability of the conical interface under simulated masticatory forces.

## Discussion:

The present *in vitro* study evaluated the micro-gap at the implant-abutment interface for three connection designs and found that external hex connections exhibited the largest micro-gaps, followed by internal hex, while internal conical (Morse taper) connections showed the smallest values both before and after cyclic loading. This trend, along with the significantly lower increase in micro-gap under loading in the conical group, suggests superior mechanical stability and a potentially better biological seal for Morse taper interfaces. Our findings are consistent with those of D'Ercole *et al.* (2022) [10], who compared bacterial microleakage in external hex, internal hex and cone Morse connections and reported significantly less leakage in Morse taper systems than in external and internal hex designs. Their conclusion that conical interfaces behave more favorably in terms of bacterial penetration supports the clinical relevance of the smaller micro-gaps observed in the present study. Similarly, Kowalski *et al.* (2023) [11] used micro-CT to assess micro-gaps and demonstrated that internal Morse cone and butt-joint connections tend to present reduced misfit compared with internal hex designs, reinforcing the concept that connection geometry critically influences micro-gap magnitude and distribution. The systematic review by Mishra *et al.* (2017) [12] further corroborates our results by summarizing that external hexagon systems show the greatest microleakage under both static and dynamic loading, whereas internal conical (Morse taper) connections generally exhibit less leakage and better stability. This aligns closely with the present study, where the external hex group demonstrated not only the highest baseline micro-gap but also the largest increase after cyclic loading, suggesting greater susceptibility to micro-movement and bacterial ingress during function. Khorshidi *et al.* (2016) [13] directly compared an 11°C Morse taper interface with a butt-joint connection and found significantly reduced bacterial leakage in the Morse taper group. Their work supports the biomechanical rationale that a conical, self-locking interface can approximate a "cold weld," thereby minimizing the effective micro-gap and limiting microbial penetration. The smaller micro-gaps seen in our internal conical group, even after simulated masticatory loading, are in agreement with this concept and highlight the potential advantages of such designs in maintaining peri-implant tissue health. Taken together, these comparative findings suggest that while no implant system completely eliminates micro-gaps, conical and mixed internal connections tend to perform better than traditional external hex designs in terms of both mechanical integrity and biological sealing. However, variations in implant systems, measurement techniques and loading protocols across studies indicate the need for standardized testing models and long-term clinical correlation. Within the limitations of this *in vitro* design, the present study adds quantitative evidence in favor of internal conical connections and underscores the importance of selecting connection geometry carefully to minimize micro-gap associated complications.

## Conclusion:

Data shows that implant-abutment connection design significantly influences micro-gap formation, with external hex showing the highest gaps and internal conical connections the lowest. Cyclic loading increased micro-gap values across all groups, but the increase was minimal in the conical interface, indicating superior mechanical stability. These findings highlight the importance of selecting advanced connection geometries to reduce micro-gap-related biological and mechanical complications in implant dentistry.
